# A review of burnout among doctors in South Africa: Pre-, during and post-COVID-19 pandemic

**DOI:** 10.4102/safp.v66i1.6002

**Published:** 2024-10-28

**Authors:** Saajida Khan, Itumeleng Ntatamala, Shahieda Adams

**Affiliations:** 1 Occupational Medicine Division and Centre for Environmental and Occupational Health Research, School of Public Health, Faculty of Health Sciences, University of Cape Town, Cape Town, South Africa; 2 Medical School, Faculty of Health Sciences, Nelson Mandela University, Gqeberha, South Africa

**Keywords:** burnout, doctors, COVID-19, South Africa, prevalence, determinants

## Abstract

**Background:**

Burnout is defined as a syndrome resulting from chronic workplace stress, which has been unsuccessfully managed. By increasing the occupational challenges faced by doctors, the COVID-19 pandemic potentiated their risk for burnout. This review aimed to determine the prevalence and determinants of burnout among doctors working in South African health facilities pre-, during and post-COVID-19 pandemic.

**Methods:**

A narrative literature review was conducted. PubMed, SCOPUS and EBSCO databases were searched for English publications until April 2024.

**Results:**

A total of 22 cross-sectional studies were included. The prevalence of burnout among doctors working in South African health facilities ranged from 4% to 84% pre-pandemic and 58.9% to 78.0% during and post pandemic, respectively. The lower value of the burnout prevalence range was higher during the pandemic. This review confirmed that individual factors (gender, age, marital status, junior status, practice setting) as well as occupational factors (workload, job control, moral distress, health system issues, job satisfaction, support at work and resilience) were associated with burnout in doctors. There was no significant association between burnout and factors related to COVID-19.

**Conclusion:**

While considerable variability exists in the prevalence of burnout among doctors working in South African health facilities, pre-, during and post-COVID-19 pandemic, the pandemic resulted in greater burnout rates. Factors associated with burnout include both individual and organisational factors, which need to be addressed to mitigate burnout.

**Contribution:**

Mitigation of burnout in South African health facilities should focus on individual-based and context-related interventional measures at an organisational level.

## Introduction

The 11th Revision of the International Classification of Diseases (ICD) defines burnout as a ‘syndrome conceptualized as resulting from chronic workplace stress that has not been successfully managed’.^1^ Burnout, is typically characterised by three dimensions including ‘feelings of energy depletion or exhaustion; increased mental distance from one’s job or feelings of negativism or cynicism related to one’s job; and reduced professional efficacy’.^1^ It is a syndrome commonly found among individuals working in human service professions and is thought to result from the emotional demands associated with these occupations.^2^ Continued exposure to occupational stress results in burnout in healthcare workers (HCWs) too, and it has been reported that medical doctors are at increased risk of burnout in comparison to other professionals.^3^

Burnout affects not only the physical health of doctors but also negatively impacts their mental wellbeing, resulting in depression, anxiety, detrimental alcohol or substance abuse and an increased risk of suicidal ideation.^4^ Burnout also affects patient care and has been associated with higher risks of medication and diagnostic errors, reduced physician-patient rapport and poor patient outcomes.^5,6^ Consequently, it has been associated with increased malpractice litigation risk.^4,5,6^ Burnout has unfavourable consequences for both organisations and health systems by reducing productivity and job satisfaction and increasing absenteeism, presenteeism and staff turnover in healthcare institutions.^4,5,6^ As such, burnout in doctors has significant consequences for society^4^ and may negatively impact the achievement of the sustainable development goal (SDG) 3 of good health and wellbeing in most countries.

The lives of HCWs across the globe have been significantly impacted upon since the World Health Organization (WHO) declared a Public Health Emergency of International Concern because of the global outbreak of COVID-19 on 30 January 2020.^7^ The spread of COVID-19 has resulted in doctors on the frontline of the health system having to contend with several challenges including high workloads, fear of infection and inadequate supply of personal protective equipment (PPE) increasing their risk of burnout.^8^ Doctors working in low- and middle-income country (LMIC) settings such as South Africa were placed at greater risk because of factors such as staff constraints, inadequate physical infrastructure, inadequate access to vaccines, shortage of medical equipment and PPE.^9,10^ On the 05 May 2023, WHO declared that COVID-19 was no longer a Public Health Emergency of International Concern while emphasising that the disease was now established and endemic in the community.^7^

Burnout has been extensively studied in physicians working in high-income countries pre-, during and post-pandemic. Systematic reviews examining burnout of doctors in the era of COVID-19 showed that the advent of COVID-19 heightened existing challenges that physicians faced such as increasing workload, which is directly correlated with increased burnout.^9,10^ Further research is, however, required to determine whether working during the COVID-19 pandemic necessarily correlated with increased burnout in doctors, particularly in South Africa, where there is a paucity of research on the prevalence and determinants of burnout in doctors working during and post pandemic. This gap in knowledge may prevent policymakers from understanding how best to mitigate burnout in doctors working in resource-limited settings such as South Africa. Therefore, this review aimed to (1) investigate the prevalence of burnout in doctors working in South African health facilities pre-, during and post-COVID-19 pandemic, (2) explore the factors associated with burnout in doctors in these settings, (3) identify preventative interventions that would successfully reduce burnout among doctors in South African health facilities.

## Research methods and design

### Study design

A structured narrative literature review was conducted on studies of burnout prevalence and determinants among doctors working in South African health facilities pre-, during and post-COVID-19 pandemic.

### Literature search strategy

For this review, a search was conducted on PubMed, Scopus and EBSCO databases to identify articles on the prevalence and determinants of burnout in medical doctors. The search used different combinations of the following key words: (((burnout) or (“Burnout, Professional”[Mesh] or “Burnout, Psychological”[Mesh])) and ((doctor or doctors or clinician or clinicians or physician or physicians) or (“Physicians”[Mesh]))) and ((“south africa”) or (“South Africa”[Mesh])) or (“South Africa”[Mesh]))) and ((“SARS-CoV-2”[Mesh] or “COVID-19”[Mesh]) or (COVID 19 or 2019-nCoV Infection or Infection, SARS-CoV-2 OR 2019 Novel Coronavirus Disease or COVID-19 Virus Infections or COVID19 or Coronavirus Disease 2019 or Severe Acute Respiratory Syndrome Coronavirus 2 Infection or SARS Coronavirus 2 Infection or 2019-nCoV Disease or COVID-19 Pandemic)). The reference lists of retrieved articles were also analysed for relevant citations, which then were included in the review. The review focussed on determining the prevalence of burnout and identifying the factors (individual, occupational and COVID-19 related) contributing to burnout, with a specific focus on articles dealing with interventions for mitigating burnout in doctors. Articles were categorised according to the timing of studies conducted, namely prior to or during and after the pandemic.

### Eligibility criteria

Inclusion criteria: (1) study design: cross-sectional or cohort study; (2) study setting: South African health facilities; (3) study population: doctors working in public and/or private health facilities; (4) primary outcome of study: burnout prevalence as assessed using a validated burnout measurement tool; (5) secondary outcome of study: factors associated with burnout; (6) studies published until 22 April 2024.

Exclusion criteria: (1) duplicates of publications; (2) studies not in English; (3) studies in countries other than South Africa; (4) studies on nurses, dentists, medical students and HCWs; (5) case reports, case series, editorials and qualitative studies; (6) research on anxiety, depression or occupational stress that did not have a specific focus on burnout.

### Data collection process and data items

The titles and abstracts of identified studies were screened, and duplicates were removed. Studies considered eligible for full-text screening were retrieved for full review. Data extracted from each paper satisfying the inclusion criteria were entered into summary tables^11^ (Table 1 and Table 2) in the following categories: (1) study author(s), (2) year of publication, (3) measurement tool, (4) study population and sample size, (5) prevalence of burnout according to measurement tool subscales and (6) prevalence of overall burnout. In the review, study characteristics, the burnout prevalence range and factors associated with burnout are reported narratively for all eligible studies.^11^

**TABLE 1:** Prevalence of burnout among doctors in South Africa pre COVID-19.

Author	Publication year	Measurement tool	Study population (*n*)	Emotional exhaustion (%)			Depersonalisation (%)			Personal accomplishment (%)			Burnout
				Low	Med	High	Low	Med	High	Low	Med	High	
Schweitzer^28^	1993	SIM	Junior doctors who graduated from two South African medical schools two and half years previously (126)	-	-	-	-	-	-	-	-	-	77.8% had experienced burnout since graduating, 52.4% were experiencing burnout in their present jobs
Peltzer^12^	2003	MBI	Doctors selected at random from 27 551 doctors registered with the HPCSA (402)	-	-	-	-	-	-	-	-	-	Mean score was 24.2 (s.d. = 10.8) for emotional exhaustion, 11.4 (s.d. = 6.7) for depersonalisation and 17.4 (s.d. = 6.8) for professional
Stodel^13^	2011	MBI	Junior doctors at Red Cross War Memorial Children’s Hospital (39)	0.0	10.0	90.0	10.0	20.0	70.0	10.0	35.0	55.0	Mean score of emotional exhaustion was 37.68
Rossouw^14^	2014	MBI	Doctors at Cape Town Metropolitan Municipality clinics and district health facilities (132)	22.0	25.0	53.0	13.0	23.0	64.0	26.0	31.0	43.0	76%
Van der Walt^29^	2015	MBI-HSS	Doctors working in the department of anaesthesiology at the University of Witwatersrand (124)	27.4	27.4	45.2	21.8	28.2	50.0	18.5	35.5	46.0	High level of burnout in 21% of Wits doctors
			Private anaesthetists attending symposium (86)	64.0	15.1	20.9	50.0	23.3	26.7	29.1	33.7	37.2	High level of burnout found in 8.1% of private anaesthetists
Sirsawy et al.^25^	2016	MBI-HSS	Registrars and medical officers at public healthcare facilities in Bloemfontein (205)	24.9	27.3	47.8	28.3	31.7	40.0	18.5	43.4	38.1	High degree of burnout found in 15.6% of participants
Rajan and Engelbrecht^17^	2018	MBI-HSS	Doctors in public sector Emergency centres in Gauteng (93)	7.5	25.8	66.7	19.3	26.9	53.8	30.1	47.3	22.6	Moderate to high risk
Liebenberg, Coetzee and Conradie^15^	2018	MBI-HSS	Doctors in district health system in Overberg and Cape Winelands (36)	11.0	33.0	56.0	11.0	14.0	75.0	14.0	42.0	44.0	81%
Zeijlemaker and Moosa^3^	2019	MBI	Registrars in School of medicine at University of the Witwatersrand (201)	14.1	19.4	66.5	5.9	19.4	74.7	52.4	25.3	22.4	84%
Balie, Branch and du Toit Lombaard^18^	2019	MBI-HSS	Obstetrics and Gynaecology registrars in teaching health facilities of the University of the Witwatersrand Medical School (47)	12.8	2.1	85.1	8.5	12.8	78.7	48.9	28.8	21.3	6.0% of respondents showed burnout in all three subgroups. There were high levels both of emotional exhaustion (85.0%) and depersonalisation (78.7%)
Coetzee and Kluyts^30^	2020	MBI-HSS-MP	Anaesthetists in public sector (189)	49.0	21.0	49.0	34.0	28.0	38.0	41.0	29.0	30.0	36.5%
			Anaesthetists in private Sector (309)	61.0	17.0	22.0	62.0	18.0	20.0	28.0	25.0	47.0	14.2%
Groenewald, van Nutgeren and Parker^16^	2020	MBI-HSS	Groote Schuur Hospital anaesthesiologists (75)	13.0	27.0	60.0	32.0	20.0	48.0	23.0	33.0	44.0	4.0% of respondents were classified as ‘burnt out’, 67.0% of respondents scored high for at least one of the components of burnout
Naidoo, Tomita and Paruk^22^	2020	MBI-HSS	Medical doctors at five KwaZulu-Natal public sector training health facilities (150)	30.0	21.3	48.7	32.7	22.0	45.3	43.3	34.7	22.0	59%
Morar and Marais^19^	2022	MBI-HSS-MP	Psychiatric registrars at the University of the Witwatersrand (33)	16.1	32.2	51.6	41.9	19.4	38.7	16.1	48.4	35.5	67.8% had scores in high category for any one of three subscales
Nazeema et al.^20^	2023	MBI-HSS	Doctors employed at Charlotte Maxeke Johannesburg Academic Hospital (327)	-	-	60.9	-	-	59.9	55.4	-	-	46.2% screened positive for burnout
Allie and Govender^23^	2023	MBI-HSS-MP	Anaesthetists at eThekwini Hospital Complex and Pietermaritzburg Metropolitan state health facilities in KwaZulu-Natal (139)	34.5	23.7	41.7	43.2	18.7	38.1	18.0	29.5	52.5	Prevalence of extreme burnout 18.7%
Mamorobela et al.^27^	2023	MBI	Doctors at the Mankweng and Pietersburg tertiary academic health facilities in Limpopo province (150)	42.0	27.0	33.0	61.0	26.0	13.0	40.0	37.0	23.0	36%

Note: Please see the full reference list of the article for more information.

SIM, Single-item Measure; MBI, Maslach Burnout Inventory; MBI-HSS, Maslach Burnout Inventory-Human Services Survey; MBI-HSS-MP, Maslach Burnout Inventory-Human Services Survey for Medical Personnel; s.d., standard deviation; Wits, University of the Witwatersrand.

**TABLE 2:** Prevalence of burnout among doctors in South Africa during and post COVID-19.

Author	Publication year	Measurement tool	Study population (*n*)	Emotional exhaustion (%)			Depersonalisation (%)			Personal accomplishment (%)			Burnout
				Low	Med	High	Low	Med	High	Low	Med	High	
Hain et al.^24^	2021	MBI-HSS	Doctors in 15 rural health facilities in KwaZulu-Natal Province (96)	22.5	19.1	58.4	23.6	16.9	59.6	48.3	24.7	27.0	68.5%
O’Connor et al.^31^	2022	SPFI	South African Orthopaedic doctors (156)	-	-	-	-	-	-	-	-	-	72.0%
Van der Merwe et al.^26^	2023	CBI	Medical registrars at the University of the Free State (60)	-	-	-	-	-	-	-	-	-	Highest median was personal scale (58.3), with 70.0% respondents scoring ≥ 50. Lowest median was patient scale (29.2), with 20.4% respondents scoring ≥ 50.
Duffton et al.^21^	2023	MBI	Frontline doctors in Tshwane public health facilities (163)	19.0	26.4	54.6	32.5	31.9	35.6	39.3	31.9	28.8	Clinical burnout was present in 58.9% and extreme burnout in 19.6%
Khan et al.^11^	2024	OLBI	Doctors in three public sector hospitals in Gqeberha (260)	-	-	-	-	-	-	-	-	-	78%

Note: Please see the full reference list of the article for more information.

CBI, Copenhagen Burnout Inventory; MBI, Maslach Burnout Inventory; MBI-HSS, Maslach Burnout Inventory-Human Services Survey; OLBI, Oldenberg Burnout Inventory; SPI, Stanford Professional Fulfilment Index.

### Ethical considerations

No ethical approval was required for the narrative review as the review is based on previously published data.

The narrative review is based on the literature review that was done for the initial study done for an MPhil thesis submission. Ethical clearance was obtained for the initial study with ethical clearance number HREC REF: 616/2021.

## Results

### Characteristics of the studies

**FIGURE 1:**
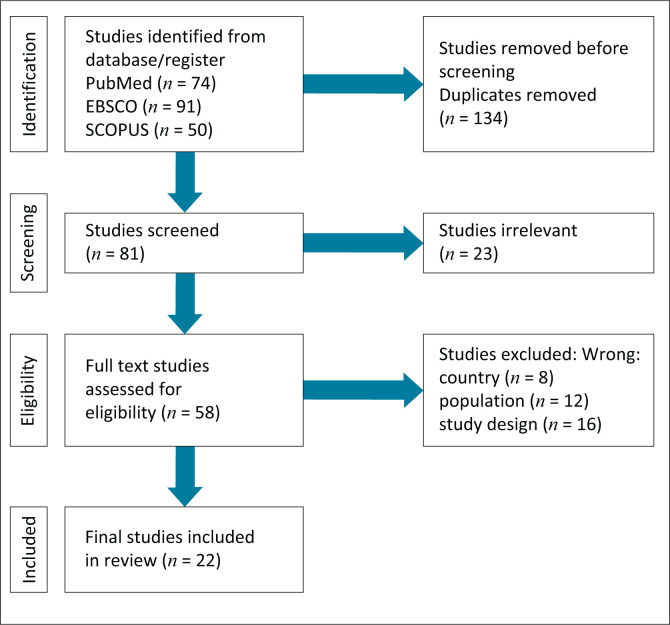
Study selection process.

Our search identified 215 possible studies. Following the removal of duplicates, a total of 81 articles were checked for screening. The study investigator screened the titles and abstracts of these studies, excluding 23 as irrelevant. The 58 remaining studies went through an assessment for eligibility, based on the predetermined criteria, which resulted in 22 studies chosen from the databases and included in the final review (Figure 1).

All included studies were cross-sectional studies assessing burnout at a single time point before 2024. Studies that form part of this review are listed in Table 1 and Table 2. Analysis of the articles identified several themes in the published research: prevalence, factors associated with burnout and prevention of burnout. The geographic distribution of studies was as follows: one study was a national study,^12^ four studies were from the Western Cape province,^13,14,15,16^ seven were from Gauteng province,^3,17,18,19,20,21^ three were from KwaZulu-Natal,^22,23,24^ while two were conducted in the Free State province,^25,26^ one each in Limpopo^27^ and Eastern Cape^11^ provinces. In three studies,^28,29,30^ the geographical location of the study was not specified as the they compared doctors either having graduated from different medical schools or doctors working in different sectors (public versus private). The age range of doctors spanned from the early 20s to > 55 years, and all studies included male and female participants. Most studies sampled consisted of consultants, medical officers, registrars and interns. However, two studies were conducted only on junior doctors^13,28^ and four studies focussed solely on registrars.^3,18,19,26^ Four studies were conducted only on doctors working in anaesthetics,^16,23,29,30^ one study focussed only on orthopaedic doctors^31^ and another on doctors working in emergency centres.^17^ Of the 22 studies reviewed, 17 were conducted pre-pandemic,^3,28,12,13,14,15,16,17,18,19,20,22,23,25,27,29,30^ and 5 were conducted during and post-pandemic.^11,21,24,26,31^ The articles included in this narrative review will be discussed by theme and chronologically based on their period of study with reference to the pandemic. Most of the studies focussed on the prevalence of burnout and determinants of burnout, and some studies included interventions to mitigate burnout among doctors.

### Measurement tools for burnout

Among the 22 included studies, standardised questionnaires were used to measure burnout prevalence among doctors. The majority (81%) assessed burnout using the Maslach Burnout Inventory (MBI) or one of its adapted or modified versions such as the MBI-Human Services Survey (MBI-HSS) or the MBI-Human Services Survey for Medical Personnel (MBI-HSS-MP). A single-item burnout measure was used in one study.^28^ The other three studies used either the Copenhagen Burnout Inventory (CBI), the Oldenburg burnout Inventory (OLBI) or the Stanford Professional Fulfilment Index (SPFI) to report outcomes.^11,26,31^

### Burnout prevalence

#### Burnout prevalence pre-COVID-19 pandemic

As indicated in Table 1, prior to the pandemic, the prevalence of burnout in South African doctors ranged from as low as 4.0% in Groote Schuur Hospital anaesthesiologists^16^ to as high as 84.0% in registrars at the University of the Witwatersrand^3^ throughout the review period. As early as 1994, Schweitzer^28^ found a 77.8% burnout rate among young doctors who had graduated from South African medical schools two and a half years previously.

#### Burnout prevalence during and post COVID-19 pandemic

During and post COVID-19 pandemic, the prevalence of burnout among doctors working in South African health facilities ranged from 58.9% in frontline doctors working in Tshwane public health facilities^21^ to 78.0% among doctors working in Gqeberha public sector hospitals^11^ (Table 2).

### Factors contributing to burnout in doctors

**FIGURE 2:**
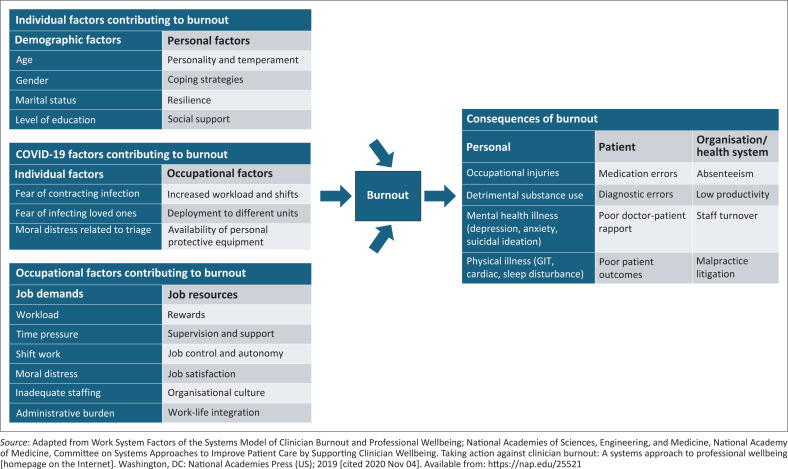
Interplay between factors contributing to burnout and consequences of burnout.

The studies reviewed described various factors, both individual and occupational, associated with burnout in doctors working in South African healthcare settings (Figure 2).

#### Individual (host) factors contributing to burnout in doctors

As indicated in Figure 2, burnout occurs because of the interaction between occupational and individual factors.^4^ Individual factors associated with burnout include demographics, personality type and level of social support.^4,11^

Sociodemographic characteristics associated with burnout in doctors include age, gender, race, marital status, level of education, occupational rank and years employed in the post.^5,6,11^ Local evidence^15,29,30,20^ suggested that burnout was more prevalent among younger doctors, with the risk of burnout decreasing with age. Rajan et al.^17^ indicated that depersonalisation was higher in respondents less than 40 years of age. Conversely, other South African studies^3,19,27^ reported no significant association between burnout and age. In this review, a considerable number of studies concluded that *female* doctors had a greater prevalence of burnout.^12,16,18,24,25,29^ However, gender was not a consistent independent predictor of burnout as these findings were inconsistent with other studies, which found no associations linking gender to burnout.^3,11,17,19,21,26,27^ While race is a social construct, with no biological basis, most studies found no association between burnout and *race*. In their national study, Peltzer et al.^12^ described that white doctors experienced more burnout symptoms than doctors of colour, and this was confirmed recently by Nazeema et al.^20^ who found burnout associated with Caucasian race in doctors in Johannesburg. Although *single status* and *being the parent of young children* appeared to be a risk factor for burnout in doctors elsewhere,^6^ local studies^15,26,27^ negated any association between relationship status and burnout. Of the studies reviewed, only O’Connor et al.^31^ described that burnout was associated with being the parent of young children.

*Occupational rank* was found to be associated with burnout in some South African studies. Burnout was associated with being an intern^11,22,24^ or community service medical officer,^11,24^ a registrar^31^ or being both intern or registrar.^20^ With regard to *practice setting*, some studies analysed the difference in burnout prevalence in doctors working in public sector health facilities in comparison to the private sector. While O’Connor et al.^31^ found no association between practice sector in orthopaedic surgeons, both van der Walt et al.^29^ and Coetzee and Kluyts^30^ found that public sector anaesthetic doctors exhibited a greater prevalence of burnout. Concerning different *specialities*, Nazeema et al.^20^ confirmed that specialities at greatest risk of burnout were frontline specialities, such as internal and emergency medicine. In contrast, other studies found no significant association between specialities and burnout levels.^3,11,27^

The presence of *mental health conditions*, such as depression and anxiety in public sector doctors, was noted to vary across burnout studies conducted in South Africa. Duffton et al.^21^ and Naidoo et al.^22^ showed that depression was present in 21% of respondents, and burnout was significantly associated with screening positive for depression and anxiety symptoms, respectively. Rossouw et al.^14^ and Hain et al.^24^ similarly found a high prevalence of depression of 30% and 35%, respectively. Interestingly, Duffton et al.^21^ also noted double the prevalence of anxiety compared to both Rossouw et al.^14^ and Hain et al.^24^ In some of the studies reviewed, substance use to control work-related stress was significantly associated with burnout. O’Connor et al.^31^ found that the use of alcohol as a coping mechanism was associated with an increased likelihood of burnout, which concurred with the findings of Khan et al.^11^ where burnout was significantly associated with using alcohol to manage work-related stress.

#### Occupational factors contributing to burnout in doctors

According to the Job Demand-Resources model, in any job, two sets of variables involving occupational factors are recognised: job demands and job resources (Figure 2).^32^ Burnout can be predicted when increased effort is required to meet job demands (increased workload, shift work, moral distress or injury, inadequate staffing and time pressure) because of a lack of job resources (rewards such as salaries and career development, job support, job control and autonomy as well as job satisfaction).^3,11,32^

Quantitative job demands (such as workload and time constraints) and qualitative job demands (such as role conflict and role ambiguity) are related to burnout.^4^ *Workload* was found to be the most significant cause of burnout in doctors in some reviewed studies.^13,16^ The number of hours worked, workload and working conditions were ranked as the important burnout contributing factors by some local studies.^13,14,30^ Interestingly, both O’Connor et al.^31^ and Khan et al.^11^ found no association between burnout and workload in studies conducted during the pandemic. The hierarchical nature of the medical profession does not allow all doctors – *job control* – to participate in the decision-making process, resulting in distress.^3,29^ Groenewald et al.^16^ indicated that registrars had a lower perception of control than consultants. Furthermore, *time pressure* results from the lack of time to complete onerous clinical tasks as well as teaching obligations and continuing medical education with the resultant completion of work after hours.^6,29^ Compliance with these requirements encroach on time spent on personal activities or with family, and Morar and Marais^19^ confirmed that *poor work-life balance* was significantly associated with burnout. *Work inefficiencies* result from increased administrative burden because of constant documenting of clinical tasks that potentiate burnout, especially when inefficient work processes do not contribute meaning in doctor’s work activities.^12,22,29^ Being subjected to unreasonable workload and demands that conflict with their moral and ethical commitments to care for their patients, increases the *moral distress* in doctors working in South African health facilities.^29^ In their study, O’Connor et al.^31^ found that screening positive for moral injury placed respondents at increased risk of burnout. Regarding job resources, Khan et al.^11^ confirmed that low *job support* was significantly associated with burnout and Naidoo et al.^22^ and Hain et al.^24^ showed a lack of clinical supervisor support and management support to be significantly associated with burnout. Finding meaning and purpose in work can be protective against burnout^9^ as confirmed by Khan et al.^11^ who noted an inverse association between job satisfaction and burnout.

#### COVID-19-related factors contributing to burnout in doctors

The COVID-19 pandemic increased the burden on doctors by increasing the stress associated with the unavailability of PPE, fear of contracting the infection and risk of infecting loved ones.^3,8,11^ Consequently, these occupational stressors brought about by the pandemic, could result in increased burnout,^8^ as indicated in Figure 2. Although five of the studies reviewed were conducted during and post-COVID-19 pandemic,^11,21,24,31^ only three of them specifically explored the association between COVID-19 factors and burnout.^11,21,31^ Contrary to expectations, Khan et al.^11^ reported that there was no association between burnout and provision of PPE or other factors associated with COVID-19. Similarly, Duffton et al.^21^ noted that the impact of COVID-19 on frontline doctors in Tshwane public health facilities did not demonstrate higher levels of burnout during the pandemic. O’Connor et al.^31^ noted contradictory results as respondents felt that the concurrent experience of the pandemic at the time of the survey reduced their experience of burnout.

### Prevention of burnout among doctors in South African hospitals

To prevent burnout among doctors, studies advocated organisation-directed interventions aimed at fixing the job, by limiting the incidence of new cases through the elimination or modification of occupational stressors.^4,5^ Rossouw et al.^14^ and Naidoo et al.^22^ recommended reducing workload by implementing changes in work schedule, instituting more rest breaks and modification of shift work or overtime systems. Zeijlemaker and Moosa^3^ and Liebenberg et al.^15^ encouraged participation in decision-making and improving professional autonomy (with respect to work schedules, working hours, annual leave) to reduce job demands and improve job control. Increased supervisory and peer support at work was proposed to improve job resources^11,14,22^ with a special recommendation of retaining senior doctors with increased experience to supervise and support younger colleagues to mitigate burnout.^14^

To reduce the prevalence of burnout, few studies focussed on strengthening resilience. Rossouw et al.^14^ and Van der Merwe et al.^26^ reported that the median score of respondents on the Connor-Davidson Resilience Scale corresponded with high self-perceived levels of resilience. Khan et al.^11^ showed resilience to be protective against burnout. Many of the reviewed studies recommended increasing resilience among doctors with mentoring programmes (to develop life skills such as boundary setting, finding work-life balance and self-care) and also proposed mindfulness-based stress reduction programmes and peer collaboration.^11,15,22,26^ Naidoo et al.^22^ posited that improving resilience in doctors would increase their levels of personal accomplishment and job satisfaction to reduce burnout.

The studies reviewed recommended treating employees who were already diagnosed with burnout with individual-directed interventions such as cognitive behavioural therapy, mindfulness and self-understanding to reduce residual deficits following burnout.^3,33^ Implementation of cognitive behavioural techniques was endorsed to challenge negative thoughts and improve job competence, communication techniques and coping skills, allowing for return to work.^11,31^ The use of mental health initiatives such as PPE for doctors’ emotional and mental health was advocated to reduce occupational stress and prevent burnout.^11^ Organisational wellness initiatives and self-care skills training were suggested to promote physical health, self-compassion and harm avoidance as interventions to reduce burnout.^3,15,22,34^

## Discussion

This study showed that although the burnout levels among doctors working in South African health facilities varied, it was significantly high, both pre-, during and post-pandemic. The substantial variability in burnout prevalence estimates reported in the studies reviewed, resulted from the heterogeneity of tools used to objectively assess burnout in various studies, disparate study populations involving various categories of doctors, as well as diverse contexts in which the participants lived and worked.^11,33^ These differences may reflect the use of different survey instruments, selection and recall bias, social desirability bias and a healthy worker effect, which introduce challenges when comparing burnout rates among doctors across different provinces.^9,33^ As a result of burnout being defined as an occupational phenomenon^1^ rather than a clinical diagnosis, it has been described mainly as a research domain topic with the lack of a standardised measurement tool contributing to the variability of burnout prevalence estimates.^10^ Dugani et al.^9^ posited that the variability in burnout prevalence between countries was a result of disparate resources available in health systems of LMICs, such as South Africa where limited resources, understaffing and poor working conditions may aggravate burnout risk. The lower burnout prevalence within some studies may be because of these doctors having greater experience of working in adverse conditions with limited resources, which results in greater resilience and less burnout.^8,35^

The review showed that the lower value of the burnout prevalence range was higher during the pandemic, which was consistent with prior reports from the United States (US) where the burnout prevalence increased 26% since 2018.^36^ Although burnout increased from 47% in 2021 to 53% in 2023 in physicians from the US, only 8% of these doctors felt that the stress of treating COVID-19 patients was the primary cause of their burnout.^36^ Similarly, in this review, while some factors related to COVID-19 infection and workplace interventions were associated with burnout, none of the associations were statistically significant. With the exception of increased moral distress reported by O’Connor et al.,^31^ specific COVID-19 factors did not appear to be associated with the increased prevalence of burnout in this review. This may be because of the period of data collection affecting burnout levels, as Morgatini et al.^8^ noted higher burnout levels in countries experiencing peaks of COVID-19 infection, during the period of data collection, compared with countries where COVID-19 was declining. The studies listed in Table 2^11,24,21,26,31^ were conducted in November 2020–June 2021, August 2021–November 2021 and April 2022–May 2022, which corresponded with the second, third and fifth waves of the pandemic in South Africa. The contributions of different phases or waves of the COVID-19 pandemic on burnout prevalence were noted in systematic reviews,^37,38^ with the early pandemic period associated with a higher prevalence of burnout compared to the late pandemic period as increased knowledge and preparedness reduced fear, and the occupational stressors had changed from isolation and the lack of PPE to vaccine safety and vaccine hesitancy.^37,38^ Duffton et al.^21^ postulated that the restrictive lockdown and alcohol ban implemented in South Africa during the pandemic reduced the volume of trauma cases, which normally burdened local health facilities, reducing occupational stress and burnout risk. Consequently, these differing stressors during waves may account for the variability of prevalence findings in Table 2.

When comparing the studies that used the MBI to measure burnout, it is interesting to note that percentages pertaining to the emotional exhaustion (EE) and personal accomplishment subscales are relatively similar across studies in South Africa, except for a few studies^3,13,16,18,20^ that had higher EE levels. Some of these studies were conducted at academic centres where the greater academic expectations on doctors may have contributed to higher EE. In contrast, this review found great variability between studies in depersonalisation levels. The higher depersonalisation levels found in some studies^3,13,14,18^ are concerning, as increased depersonalisation perpetuates the development of a callous and cynical attitude, which, in doctors, may compromise patient care in an already litigious environment.

This review found that younger age contributed to burnout in South African doctors,^20,29,30^ which correlated with other systematic reviews^37,38^ that reported junior doctors to be more vulnerable to burnout because of their lack of experience and resultant inability to manage system factors. While gender was not found to be a consistent independent predictor of burnout, given the current feminisation of medicine and the increased proportion of female doctors qualifying, the impact of gender on burnout requires consideration because having to balance more family responsibilities as mothers, may potentiate work-life imbalance in female doctors.^6,16^ While burnout has been associated with substance use and psychiatric diagnoses in this review, the bidirectional relationship between burnout and depression makes it difficult to determine causality because of the cross-sectional nature of these studies. Similarly, it is not clear whether alcohol use preceded or followed burnout.^11^ However, it is possible that respondents may have used alcohol as a maladaptive coping mechanism.^11^ As a result of the conflicting evidence in the literature regarding the associations linking social and demographic factors to burnout, it has been suggested that the origins of burnout emanate from contextual factors that should be quested at the workplace.^3,4,11^ Although many studies suggested that specialities at greatest risk of burnout were frontline specialities,^37,38^ it is interesting to note that in this review, four studies^16,23,30,29^ dealt specifically with burnout in doctors practising anaesthetics and found that their prevalence of burnout was high despite the differences in geographical location. To explain this preponderance, it has been posited that because South African public health facilities experience a shortage of anaesthesiologists, the anaesthesia workforce is overwhelmed by the increased clinical demands in an under-resourced workplace.^23^ The finding that public sector anaesthetic doctors exhibited a greater prevalence of burnout in comparison to doctors in the private sector is also concerning as this may negatively impact the retention of doctors within the resource-constrained public healthcare system.^24,29,30^

This review aligns with global studies,^6,10,37,38,39^ which propose that the high levels of burnout are related to occupational factors such as the heavy patient workload with poor doctor-patient ratio that increases job demands^14,19,24^ and therefore recommend adjustment of work schedules. This review has shown that the hierarchical cultures found in most South African health facilities result in structured workplaces with strict procedures, formal rules and rigid policies with little job control and autonomy, which increase burnout by reducing job satisfaction.^3,4,5,11^ The prevailing culture within the medical profession is not characterised by high levels of mutual support and the absence of supportive managerial supervision at an organisational level in South African health facilities appears to be related to burnout.^11,22,24^ Poor help-seeking behaviour reported among doctors is perpetuated by the stigma and discrimination facing those doctors who disclose physical, mental or substance abuse problems.^6,11,12,22,29^ To address this unsupportive organisational culture, investment is required in adopting strategies of optional resilience training, mentoring and offering supervisory support to doctors, especially the young doctors or those doctors reporting mental health or substance use disorders.^11,13,22,39^ However, caution must be taken to avoid focussing more on resilience and wellness rather than managing organisational factors, else only the symptoms of burnout rather than the cause would be treated.^11,34,39^ Therefore, to balance both organisational and personal responsibilities in the aetiology of burnout, organisations need to complement individual-based interventions while addressing workplace stressors and organisational culture that compromise resilience and engagement.^4,34,39^

The variability in burnout estimates reported in this review is consistent with those reported in international systematic reviews on the prevalence of burnout among doctors conducted both pre- and post- pandemic.^9,10,33,35,37,38,39^ The evidence provided by this study reflects the diversity in the criteria used by the South African studies to define and measure burnout and highlights the need for uniformity in the measurement and definition of burnout to mitigate the heterogeneity. Notwithstanding the variation in burnout estimates, the evidence provided by this study identifies the individual and occupational factors that remain a challenge for doctors and requires that organisations and policymakers consider the context within which medical doctors work.

### Limitations

This review had several limitations. These include a small number of local articles that had inconsistent methodologies. Additionally, the studies applied different assessment tools, and even when the same tool was used, there was no consensus on the burnout rate. This lack of consensus with resultant variability in burnout prevalence reports limits the ability to make reliable comparisons between studies. A further limitation is that the current review includes studies conducted during different phases of the pandemic, which contribute to the heterogeneity of the data.

### Implications and recommendations

The COVID-19 pandemic has exposed the systemic inefficiencies in already overburdened healthcare systems of many countries. In many South African public health settings, because of existing resource constraints and understaffed health facilities, the burden on doctors has been severe. While COVID-19 presented doctors with unprecedented workplace challenges, the pandemic also presents an opportunity for stakeholders and policymakers to make fundamental changes in the healthcare system to ensure a sustainable response to mitigate burnout in doctors. To mitigate the variability in the prevalence of burnout reported in previous studies and to render a fair comparison of results, it is recommended that further research be based on standardised, validated open-access instruments with universal cut-off scores.^11^ In future studies, a systematic review and meta-analysis of the studies, which use the same tool and cut-off points, may be done to mitigate the heterogeneity identified. Longitudinal studies investigating the effectiveness of interventions in mitigating burnout among doctors are also recommended.

## Conclusion

Burnout among doctors is a global concern, and this narrative review confirmed that the prevalence of burnout in doctors was substantial in South African health facilities prior to the COVID-19 pandemic with both individual and occupational factors contributing to burnout among doctors. As a result of the pandemic, South African doctors working in the public healthcare system were faced with additional occupational challenges, which increased burnout prevalence. Although doctors are resilient, the healthcare system and its professional regulatory bodies have a responsibility in supporting the mental health and wellbeing of doctors by targeting clinician concerns to ensure the retention of this resource. This review provides knowledge regarding the determinants of burnout among doctors working locally, pre-, during and post-COVID-19 pandemic, which will contribute to the mitigation of burnout among doctors. The significant consequences burnout has for individual doctors, patients, as well as healthcare systems makes it imperative for organisations, regulatory bodies and policy makers to mitigate burnout in doctors working in South African health facilities by addressing the factors identified in this review and implementing the proposed preventative interventions at individual and organisational levels.
